# A Review of Prognosis Model Associated With Cardiogenic Shock After Acute Myocardial Infarction

**DOI:** 10.3389/fcvm.2021.754303

**Published:** 2021-12-10

**Authors:** Jingyue Wang, Botao Shen, Xiaoxing Feng, Zhiyu Zhang, Junqian Liu, Yushi Wang

**Affiliations:** Department of Cardiology, The First Hospital of Jilin University, Changchun, China

**Keywords:** cardiogenic shock, risk stratification, myocardial infarction, prognosis, severe case

## Abstract

**Objective:** Cardiogenic shock seriously affects the survival rate of patients. However, few prognostic models are concerned with the score of cardiogenic shock, and few clinical studies have validated it. In order to optimize the diagnosis and treatment of myocardial infarction complicated with cardiogenic shock and facilitate the classification of clinical trials, the prognosis score model is urgently needed.

**Methods:** Cardiogenic shock, severe case, prognosis score, myocardial infarction and external verification were used as the search terms to search PubMed, Embase, Web of Science, Cochrane, EBSCO (Medline), Scopus, BMC, NCBI, Oxford Academy, Science Direct, and other databases for pertinent studies published up until 1 August 2021. There are no restrictions on publication status and start date. Filter headlines and abstracts to find articles that may be relevant. The list of references for major studies was reviewed to obtain more references.

**Results and Conclusions:** The existing related models are in urgent need of more external clinical verifications. In the meanwhile, with the development of molecular omics and the clinical need for optimal treatment of CS, it is urgent to establish a prognosis model with higher differentiation and coincidence rates.

## Introduction

Cardiogenic shock (CS) is the most serious complication of acute myocardial infarction (AMI). It is characterized by low systemic perfusion caused by cardiac pump failure, often leading to multiple organ failure and severe internal environment disorder, with rapid disease change and poor prognosis. Even after early revascularization, the mortality rate of patients with AMI complicated with CS is still as high as 40–50% (1). Many factors influence the prognosis of CS, including gender, age, acute anterior myocardial infarction, gastrointestinal bleeding, history of stroke, and kidney damage (2, 3). If coronary blood flow cannot reach thrombolysis in myocardial infarction (TIMI) level 3 after percutaneous coronary intervention (PCI), the prognosis of patients is poor (4). AMI is the main cause of CS, and a comparative study with non-infarct-related CS found that AMI is an independent risk factor for death from CS (5). Therefore, infarct-related CS is the most critical state of CS, and timely and accurate risk stratification for this type of CS is helpful to guide clinicians to formulate appropriate treatment plans.

With the rapid development of critical medicine, a series of methods to evaluate the severity of critical diseases have been produced, and its advantages are as follows. (1) Doctors assess the patient's condition and prognosis, measure the treatment effect dynamically, and adjust the treatment plan in time. (2) Refine the right to know about the family members of patients. (3) Guide the allocation and utilization of resources in the ward. At present, there are many scores on the prognosis of severe cases of myocardial infarction. These are mainly divided into non-specific and specific evaluation systems. Non-specific evaluation systems include the Acute Physiological and Chronic Health Score (APACHE) (6) and the Simplified Acute Physiological Score (SAPS). Both have been proven in the assessment of the prognosis of patients with myocardial infarction (7–9). Specific scoring methods for the cardiovascular system include the TIMI risk score, Primary Angioplasty in Myocardial Infarction (PAMI) score, and Zwolle. These scores have been used in most studies to evaluate the prognosis of patients with myocardial infarction or coronary intervention and have achieved good results (10, 11). French scholars have shown that SAPS II score, cardiac function index, and mean arterial pressure are prognostic factors of CS in patients (12). At the early stage, there were some risk scores for CS (5, 13, 14), but these were small studies, and most patients failed to undergo emergency PCI due to limited early medical conditions. With the progress of medical technology, several studies have shown that emergency PCI revascularization can significantly benefit patients with CS (15, 16). Among these scores, some scoring models were created too early. Other projects were too big, complicated, and inconvenient to implement. Some had no specific disadvantages for CS. Therefore, these scores may have a variety of biases and have limited significance for current clinical guidance.

The objective of this study was to review a variety of studies related to the scores and external validation of CS after myocardial infarction for comparison and to make relevant predictions.

## Diagnostic Criteria and Etiology of CS

The diagnostic criteria for CS are hypotension (systolic <90 mmHg or requiring vasoactive drugs to maintain systolic blood pressure ≥90 mmHg) and signs of organ hypoperfusion (central nervous system symptoms including coma or disturbance of consciousness or even loss of consciousness, oliguria, cold, clammy skin and limbs, and arterial lactic acid >2 mmol/L). Other clinical indicators were included, such as a decrease in cardiac index (CI <1.8 or <2 min/m^2^ with cycle support) or an increase in left ventricular filling pressure (pulmonary artery wedge pressure >15 mmHg) (1). The incidence of AMI combined with CS is 5–10% (1). A registered study in the United States showed that 29% of patients with AMI complicated with CS developed shock upon admission, and 71% developed shock during hospitalization (17). CS is also the most common cause of AMI death in hospitals (17). Common causes of CS include AMI combined with severe mechanical complications, severe valvular heart disease, malignant arrhythmia, explosive myocarditis, and cardiomyopathy. The analysis of CardShock showed that the most common cause of CS was acute coronary syndrome (81%; *n* = 177). Non-acute coronary syndrome etiology accounted for the remaining 19% (*n* = 42). The majority of acute coronary syndrome (ACS) patients (*n* = 148; 68% of the patients) had STEMI, and 19 patients (9%) had mechanical complications after myocardial infarction, including six cases of papillary muscle rupture, 10 cases of ventricular septum rupture, and three cases of left ventricular free wall rupture. Non-ACS etiologies mainly included chronic heart failure (11%), valvular and other mechanical etiologies (6%), stress cardiomyopathy, and myocarditis (2%) (5).

## IABP-SHOCK II Score: Intra-aortic Balloon Counterpulsation-Shock II Score

The IABP-SHOCK II risk score was based on data from a multicenter, randomized, and controlled IABP-SHOCK II trial. Six variables, including age, previous history of stroke, admission blood glucose, serum creatinine, blood lactic acid level, and coronary blood flow after PCI, were used to predict the short-term death risk of AMI with CS after emergency PCI (18, 19).

The six variables of risk score specifically included: Age >73 years (1 point), previous history of stroke (2 points), admission blood glucose >10.6 mmol/L (1 point), blood creatinine >132.6 μmol/L (1 point), blood lactic acid >5 mmol/L (2 points), and TIMI coronary blood flow < grade 3 (2 points) after PCI. The total possible score was 9, where 0–2 represented the low-risk group, 3–4 represented the medium-risk group, and 5–9 represented the high-risk group. Pöss et al. (18) showed that the 30-day mortality of low-risk patients was lower than that of medium- and high-risk patients (23.8 vs. 49.2 vs. 76.6%, *P* < 0.01). The limitations of IABP-SHOCK II risk scoring are as follows. (1) The data were limited to Western populations. (2) The risk score was calculated from a cohort study of 480 patients and validated in a relatively small sample size of 137 and 98 patients, respectively. (3) Estimation of TIMI blood flow in this scoring system was provided by the investigator and not reviewed by core laboratory personnel, so it may be biased. (4) Data were missing in patients who did not undergo coronary angiography (e.g., non-acute coronary syndrome etiology). Therefore, this score is only applicable to patients with CS caused by ACS. (5) Age is one of the scoring variables, automatically assigning a score to older people. This may contribute to higher scoring levels and less dispersion of scores in older adults, potentially reducing the predictive power of risk models. However, it is worth emphasizing that the calculation of the risk score was derived from the IABP-SHOCK II study, which is currently the largest randomized controlled clinical trial on the placement of AMI with CS into IABP, with high credibility (18). Moreover, the internal validation in the IABP-SHOCK II database and the external validation in another database showed a good differentiation and calibration degree.

Five of the six variables in the IAPB-SHOCK II were included in the risk scores of CS-related, such as age (20), blood glucose (21, 22), renal function (14, 23, 24), lactic acid (25) and TIMI blood flow in the coronary artery after PCI (20). Previous studies (26, 27) have confirmed that AMI and stroke have a close correlation. For specific scoring details, see Table 1.

**Table 1:** Catalog of IABP-SHOCK II score.

**Six variables**	**Score**
Age >73 years	1
Blood glucose >10.6 mmol/L	1
Serum creatinine >132.6 μmol/L	1
Lactic acid >5 mmol/L	2
TIMI blood flow after stenting <3	2
History of stroke	2

*Pöss et al. (18). Risk stratification: 0–2 scores in the low-risk group; 3–4 scores in the medium-risk group; 5–9 scores in the high-risk group.

## CardShock Score

The CardShock Cohort Study (5) is a European perspective, observational, multicenter, and transnational study from October 2010 to December 2012. The study included patients with an ACS and non-ACS etiology within 6 h of diagnosis of CS. Exclusion criteria were arrhythmias that cause significant changes in hemodynamics and shock after cardiac or non-cardiac surgery. The CardShock score included seven items: age >75 years (1 point), obnubilation (1 point), previous myocardial infarction or after bypass surgery (1 point), ACS (1 point), left ventricular ejection fraction (LVEF) <40% (1 point), blood lactic acid 2–4 mmol/L (1 point), blood lactic acid >4 mmol/L (2 points), glomerular filtration rate 30–60 mL/(min·1.73 m^2^) (1 point), glomerular filtration rate <30 mL/(min·1.73 m^2^) (2 points). The total possible score is 9, with 0–3 being low risk, 4–5 being medium risk, and 6–9 being high risk. The CardShock score showed excellent differentiation in predicting the risk of in-hospital death in patients with CS (AUC = 0.85) (Table 2). Hongisto et al. (28) collected 219 patients from the CardShock study and stratified 33 older patients, including one case in the low-risk group, 14 cases in the medium-risk group, and 13 cases in the high-risk group, and obtained an AUC curve area of 0.75. However, this study has limitations. Although the number of patients in the prospective CardShock study is reasonable, the proportion of older people is limited, resulting in the statistical uncertainty of the comparison between groups. This is also a common problem in many studies on CS (29). Miller et al. (30) collected 510 patients with CS from tertiary hospitals in Alberta, Canada. The IABP-SHOCK II score and CardShock score were verified and compared in the CS population. Both showed general differentiation, but in the subgroup of ACS-induced CS, the differentiation of the two scores showed an increasing trend. In the validation study of Rivas-Lasarte et al. (31), it was also confirmed that the IABP-SHOCK II score and CardShock score had a good predictive effect on the risk of nosocomial death in patients with AMI and CS. In this study, it was found that both had a relatively accurate predictive ability for the risk of in-hospital death in patients with AMI and CS.

**Table 2:** Catalog of CardShock score.

**Seven variables**	**Score**
Age >75 years	1
Previous myocardial infarction or after bypass surgery	1
Pathogenesis: acute coronary syndrome	1
Previous myocardial infarction or after bypass surgery	1
LVEF <40%	1
Lactic acid (mmol/L)	
<2	0
2–4	1
>4	2
Glomerular filtration rate (CKD-EPI) (min·1.73 m^2^)	
>60	0
30–60	1
<30	2

*Harjola et al. (5). Risk stratification: 0–3 scores in the low-risk group; 4–5 scores in the medium-risk group; 6–9 scores in the high-risk group.

## Scoring System of Severe Cases

Kellner et al. evaluated 45 patients with AMI-CS (11) comparing the role of the APACHE II, APACHE III, Elebute Stone, Sequential Organ Failure Assessment (SOFA), and SAPS II scoring systems in predicting mortality in patients with CS complicated by AMI, and found that the scores of APACHE II, APACHE III, SAPS II and SOFA of dead patients were higher than those of living patients. These results suggested the SAPS II, APACHE III, and APACHE II scores at diagnosis and maximum may help predict the high probability of survival in patients with CS and AMI. Therefore, this paper mainly introduces and compares SAPS and APACHE scores.

### APACHE Score

The Acute Physiological and Chronic Health Status Evaluation (APACHE) system is an objective system used to evaluate the severity and predict the prognosis of patients with various critical diseases. It is the most widely used and authoritative scoring method in the world at present. At present, it includes types I, II, and III. APACHE I was first proposed by American scholars in 1981. However, due to its large number of parameters, complex data collection, the simple evaluation of chronic health conditions, and inconvenient and inaccurate clinical use, APACHE I is rarely used. APACHE II was proposed in 1985. APACHE II is composed of acute physiology score (APS), age score, and chronic health score (CHS), with a score of 0–71. Due to its scientific nature and objectivity, it has been widely used in clinical and scientific research and can not only be used to evaluate the prognosis of group patients but also has a certain value in predicting individual mortality. The IABP-SHOCK study included 40 patients with CS after AMI treated with PCI, in which the death group II score was significantly higher than the survival group. There was a good correlation between the initial and sequential II scores and the mortality rate, which could predict the hospital death of patients well. APACHE III was updated in 1991, but due to a large number of parameters, the calculation is too complicated, and its clinical application is limited. APACHE II is the most widely used and authoritative critical illness evaluation system in severe case wards.

### Simplified Acute Physiology Score

In 1984, Le Gall et al. (32) using APACHE II, collected data in the first 24 h after admission to ICU and came to the following conclusions. A prognostic model consisting of a physiological score, age, and mechanical ventilation was used to evaluate the mortality of patients with severe cases. In 1993 (9), SAPS II adjusted the weights of various parameters by logistic regression models in addition to the adjustment of physiological variables and included the type of admission (regular surgery, non-regular surgery, or internal medicine). Three basic disease variables (acquired immunodeficiency syndrome, metastatic cancer, and hematologic malignancies) and six variables were also identified, and a formula for calculating the predicted case fatality rate was provided.

## Cardiovascular System-Specific Scoring Methods

### TIMI Risk Score, PAMI Score

TIMI risk score is widely used for risk stratification of patients with chest pain, which clinical workers are totally familiar with, so this part will mainly introduce the PAMI score. The PAMI score, established by Addala et al. (33) in 2004, was derived from the Primary Angioplasty in Myocardial Infarction (PAMI) study, which was the first clinical trial database score generated from patients undergoing interventional STEMI treatment. In this study, 3,252 patients were enrolled. The score items were generated by logistic regression, including age, systolic blood pressure, heart rate, Killip scale, anterior wall myocardial infarction, or Left bundle brunch block (LBBB). The score range was 0–15, which was a good predictor of mortality 6 months after STEMI intervention. The area under the ROC curve was 0.77 and verified in the real world. The advantage of the PAMI score was that the data were readily available and did not include coronary angiographic features.

### Zwolle Score

The Zwolle score was published in the journal “Circulation” by De Luca et al. (34) in 2004. It was derived from a study of 1,791 STEMI patients receiving interventional therapy, including three vascular lesions, final blood flow TIMI grade, Killip grade, age, anterior wall myocardial infarction, and ischemia time, with scores of 0–16. Currently, the number of studies involving the Zwolle score is relatively small, and most of them focus on early discharge screening of patients with myocardial infarction. For example, Lim et al. demonstrated that Zwolle score stratification could identify low-risk patients for early discharge (35). However, some studies (36, 37) have shown that adding creatinine, BNP, and proBNP variables can improve the value of this score in identifying low-risk patients for early discharge. At the same time, although this score includes the features of coronary angiography, the proportion of Killip grades of heart function is relatively high, which may weaken the ability to distinguish the rates of disease and death of patients with existing CS. Therefore, this score is rarely used for reference or comparison in clinical and external verification at home and abroad.

## Cardiogenic Shock Special Scores

### Early Risk Stratification

The prediction model is based on a retrospective study. Garcia-Alvarez et al. (20) included continuous patients with myocardial infarction complicated with CS from July 2001 to December 2007 and collected clinical, hemodynamic, and echocardiographic data. According to the previous data of influencing factors of CS (38–43), multi-factor analysis was carried out, and the results were as follows. Age >75, left coronary artery occlusion, LVEF <25%, and TIMI blood flow after PCI are the most important prognostic factors. The total possible score was 4 points, categorized into a high-risk group (≥2 points), a medium-risk group (1 point), and a low-risk group (0 points). The 1-year survival rates were 83, 19, and 6%, respectively (Table 3). The advantage of this predictive model is that it is simple and easy to perform, such as in the catheterization laboratory, and a simple risk score can be quickly provided to effectively estimate prognosis regardless of location. The predictive model also has some limitations. First, it is a single-center study, and patient inclusion is done in a tertiary hospital that receives critically ill patients from other local hospitals. As a result, there may be too many high-risk cases, and the data from patients who die before reaching the catheterization laboratory are excluded from the study. Second, other variables that might be related to patient survival after PCI, such as ST-segment regression or myocardial color grading, were not studied in this study. Finally, there may be information deviation in the hemodynamics and cardiac ultrasound data recorded after surgery.

**Table 3:** Early risk stratification.

**4 variables**	**Risk score**
Age >75 years	1
Left main coronary artery occlusion	1
TIMI grade <3 flow after PCI	1
LVEF <25%	1

*Garcia-Alvarez et al. (20). Risk stratification: low-risk group (0 score), medium-risk group (1 score), high-risk group (≥2 scores).

### The Save-Score and the Encourage Mortality Risk Score Related to ECMO

Schmidt et al. (44) analyzed and summarized data from the International Registry of Extracorporeal Life Support Tissue from January 2003 to December 2013 of patients with refractory CS who were treated with venous and arterial ECMO. In this study, the area under the receiver operating characteristic curve (ROC) of the SAVE-score was 0.68 (95% CI 0.64–0.71). The score contains 12 items (Table 4). The risks were divided into class I (SAVE-score >5), class II (SAVE-score from 1 to 5), class III (SAVE-score from −4 to 0), class IV (SAVE-score from −9 to −5), and class V (SAVE-score ≤ −10). The corresponding survival rates were 75, 58, 42, 30, and 18%, respectively. In 161 Australian patients, external validation of the SAVE-score showed a good AUC (AUC = 0.90) and better differentiation than the APACHE II, APACHE III, and SOFA scores. The SAVE-score may be a tool for predicting the survival of patients with refractory CS treated with ECMO. This score is the first reported in-hospital survival prediction model for ECMO treatment of CS. Its limitations were that the SAVE-score still requires further validation and investigation by other centers and the broader ECMO population.

**Table 4:** SAVE-score.

**12 variables**	**Score**
Etiology of acute cardiogenic shock (single or multiple options)	
Myocarditis	3
Refractory ventricular tachycardia/ventricular fibrillation	2
After a heart or lung transplantation	3
Congenital heart disease	−3
Others	0
Age (years)	
18–38	7
39–52	4
53–62	3
≥63	0
Weight (kg)	
≤ 65	1
65–89	2
≥90	0
Organ failure before ECMO application	
Liver failure	−3
Dysfunction of central nervous system	−3
Renal failure	−3
Chronic renal failure	−6
Time of tracheal intubation before ECMO commencement (hours)	
≤ 10	0
11–29	−2
≥30	−4
Peak inspiratory pressure ≤ 20 cm H_2_0	3
Cardiac arrest before ECMO	−2
Diastolic blood pressure before ECMO ≥40 mmHg	3
Pulse pressure difference before ECMO ≤ 20 mmHg	−2
HCO_3_ ≤ 15 mmol/L before ECMO	−3
Adds a constant value to all SAVE-score calculations	−6

*Schmidt et al. (44). Risk stratification: I > 5 scores; II 1–5 scores; III −4–0 scores; IV −9–−5 scores; V ≤ −10 scores.

Muller et al. (45) analyzed the data of 138 patients with AMI receiving ECMO treatment in two ICU wards in France (2008–2013) and obtained a score combining seven simple variables (Table 5). These variables were age, sex, BMI, Glasgow coma scale, creatinine, lactic acid, and prothrombin activity. These are readily available before ECMO implantation, which may help physicians communicate objective prognostic information to agents and better select ECMO candidates for patients with severe AMI. In future randomized VA-ECMO studies, it may also be used to stratify the inclusion of patients with AMI-related CS. Health-related quality of life (HRQOL), and frequency of anxiety, depression, and post-traumatic stress disorder (PTSD) could also be assessed for long-term survivors. In this study, the researchers confirmed that survival rates at 0–12, 13–18, 19–22, 23–27, and ≥28 scores were 80, 58, 25, 20, and 7%, respectively, 6 months after ECMO surgery. The area under the ROC curve (AUC) of this score (0.84 [95% CI 0.77–0.91]) was significantly better than that of SAVE, SAPS II, and SOFA scores, but this study has rarely been externally validated in clinical trials at home and abroad.

**Table 5:** The ENCOURAGE mortality risk score.

**Seven variables**	**Score**
Age >60 years	5
Female	7
BMI >25 kg/m^2^	6
Glasgow coma scale <6	6
Serum creatinine >150 μmol/L	5
Lactic acid (mmol/L)	
<2	0
2–8	8
>8	11
Prothrombin activity <50%	5

*Muller et al. (45). Risk stratification: 0−12 scores; 13-18 scores; 19−22 scores; 23−27 scores; ≥28 scores.

In conclusion, the above two models are derived from patients with CS and ECMO application and have certain limitations. Moreover, the establishment of the SAVE scoring model did not limit the etiology of CS, which may lead to the generation of a variety of information offset.

### CS4P Score

CS4P score (46) was based on the levels of liver fatty acid-binding protein (L-FABP), β-2-microglobulin (β2M), Fructose-diphosphate aldolase B (ALDOB), and SerpinG1 (IC1). Although these proteins are not heart-specific, they reflect multiple organ dysfunction, systemic inflammation, and immune activation. In terms of differentiation, CS4P was better than CardShock and IABP-SHOCK scores (Table 6). However, due to the development of proteomics, its clinical application is limited.

**Table 6:** Comparison of CS4P, CardShock, and IABP-Shock II scores (46).

	**AUC**	**HL**	**P**	**Reclassification improvement indicators**	**Threshold (Yoden)**	**Sensitivity**	**Specificity**
CardShock	0.78 (0.69–0.87)	3.29	0.914	–	0.25	0.91	0.54
IABP-Shock II	0.78 (0.66–0.90)	6.64	0.576	–	0.26	0.85	0.58
CS4P	0.83 (0.74–0.89)	13.26	0.103	–	0.36	0.83	0.75
CardShock + CS4P	0.84 (0.76–0.93)	7.25	0.509	0.49	0.47	0.77	0.84
IABP-Shock II + CS4P	0.80 (0.67–0.92)	3.44	0.904	0.57	0.35	0.85	0.68

### A New Risk Score to Predict Long-Term Cardiac Mortality

A retrospective analysis (47) was performed on enrolled AMI patients treated with primary PCI. From 1995 to 2013, 4,078 AMI patients underwent PCI. CS was present in 388 patients (10.5%) at admission. The mortality rate between different scoring risk levels is very significant (*p* < 0.001): 32% scoring risk 1 (0 points), 58% scoring risk 2 (0.5–2 points), and 83% scoring risk 3 (score >2) respectively. Score details: out of hospital cardiac arrest :0.5 points, Age >75 years: 1 point, failed primary PCI: 1.5 points. This rapid scoring tool can be used to identify patients with a significant risk of death.

### A Simple Risk Chart for Initial Risk Assessment of 30-Day Mortality

Between 2000 and 2012, a series of 544 STEMI patients who underwent direct percutaneous coronary intervention and who had cardiogenic shock were enrolled by this research (24). The mortality of STEMI patients with cardiogenic shock undergoing direct percutaneous coronary intervention can be well predicted by the risk chart at the time of admission. The risk chart uses only three variables, namely age, initial serum lactic acid and creatinine levels. The risk chart has a good accuracy in predicting the 30-day mortality of patients with STEMI cardiogenic shock who choose direct PCI, and the performance is better than the established risk score (e.g., GRACE score) commonly used for ACS patients. But there are some limits that we can't ignore. Firstly, data misses were more common among variables collected retrospectively. Secondly, this risk map is not validated in a separate dataset, which is essential. Thirdly, for patients with cardiogenic shock without STEMI and without direct PCI, care should be taken to infer the results, same to the clinical trials. Last but most importantly, some measures which reflect hemodynamics parameters, microcirculation and organ dysfunction that are added into the model will be better.

### SCAI Clinical Expert Consensus Statement

To Provide a simple solution that allows clear communication about the status of patients and allows clinical trials to distinguish patients appropriately (48), American College of Cardiology (ACC), the American Heart Association (AHA), the Society of Critical Care Medicine (SCCM), and the Society of Thoracic Surgeons (STS) endorsed a clinical expert consensus statement, describing stages of cardiogenic shock from A to E. A is “at risk” for cardiogenic shock, stage B is “beginning” shock, stage C is “classic” cardiogenic shock, stage D is “deteriorating”, and E is “extremis”. The proposed classification system is simple and clinically applicable to all areas of care from pre-hospital providers to intensive care personnel, but future validation studies will be needed to assess its effectiveness and potential prognostic significance.

## Discussion

Due to the aging population, the incidence of AMI causing CS has increased significantly in the past decade, from 6.5% in 2003 to 10.1% in 2010 (49). During the same period, however, the mortality rate dropped from 62 to 48%, probably due to significant advances in supportive treatments such as revascularization and mechanical circulation. Despite advances in the management of cardiovascular disease, the mortality rate remains high. Nearly 50% of patients died within 90 days, and clinicians are currently unable to reliably assess which patients will survive. This hinders treatment optimization, clinical progress, and clinical trial design. From the perspective of molecular mechanism, CS is not only a sudden decline of systolic myocardial function but also a multi-organ dysfunction syndrome, often accompanied by systemic inflammatory reactions with severe cell and metabolic disorders. Multiple organ failure is the source of circulating molecules, such as troponin, creatinine, and albumin (50), and circulating molecules have important value in the diagnosis of CS (51). Advances in histological and cytological techniques have provided a more comprehensive molecular profile of the clinical manifestations of systemic inflammation and heart and end-stage organ failure and have led to a shift from a single clinical electrocardiogram, cardiac color, and radiographic information to biomarker research. Previous studies have shown that cardiac troponin is closely related to the poor prognosis of patients with acute coronary syndromes, which is helpful for early identification of patients and active intervention treatment to benefit patients. The higher the troponin level, the longer the duration, and the larger the area of the damaged heart muscle, the more severe the disease. Jolly et al. (52) studied whether troponin levels could predict mortality and several cardiovascular complications such as CS. Every 10-fold increase in troponin was associated with a corresponding increase in the incidence of cardiac arrest, persistent ventricular tachycardia, or ventricular fibrillation and CS. Some studies have also suggested using troponin to evaluate the systemic effects, etiology, and complications of CS (53). Both BNP and NT-proBNP play a role in the treatment of patients with heart failure (54). However, in patients with concomitant CS, the relationship and correlation between these indicators and the disease state of patients are complex. Patients treated with an intra-aortic balloon pump in PCI for AMI with CS had lower BNP levels (55). In patients with CS, increased BNP or NT-proBNP levels may be related to left and right ventricular dysfunction (56, 57), while low NT-proBNP value (<1,200 pg/mL) is not good for predicting CS (58). However, NT-proBNP level can significantly distinguish between survivors and non-survivors (4,590 ± 1,230 vs. 14,370 ± 4,886 pg/mL, *P* < 0.05), while BNP does not show a significant difference in this respect (59). Studies have shown that NT-proBNP and interleukin-6 levels play a complementary role in predicting outcomes. Patients with IL-6 >195 pg/mL and NT-proBNP higher than the median had a 30-day mortality rate of 93.7%, while patients with lower IL-6 levels and lower NT-proBNP levels had a significantly improved survival rate (mortality rate: 26.3%) (60). A study on sepsis has shown that, contrary to previous studies, the higher the neutrophil-to-lymphocyteratio (NLR), the lower the incidence of in-hospital mortality and bacteremia. NLR at admission was an independent predictor of in-hospital mortality in patients with sepsis. Studies on cardiogenic shock showed that there was an inverted U-shaped curve between NLR and CS mortality (61). NLR appears to be an accessible and independent prognostic biomarker for patients with CS. The prognostic value of NLR was more sensitive than the percentage of neutrophils or lymphocytes but was less predictive of 30-day mortality than the SAPS score (62). Compared with the traditional severe case score, cardiovascular system-specificity score, and some CS scores, the IABP-SHOCK II score and CardShock score are more popular for researchers who evaluate the condition of patients with CS. However, at present, there are few external validation studies even involving CardShock and IABP-SHOCK II scores abroad, and most of these are retrospective studies, the results of which need to be further verified by a large number of multicenter and prospective studies. Moreover, with the development of molecular omics, such as troponin, BNP, albumin, and creatinine, and the increased diagnosis rate of CS, a prognosis model of CS that is more in line with clinical and research needs is urgently needed.

## Conclusion

The existing related models are in urgent need of further external clinical verification. In the meanwhile, with the development of molecular omics and the clinical need for optimal treatment of CS, it is urgent to establish a prognosis model with higher differentiation and coincidence rates.

## Author Contributions

JW and YW conceived the idea, conceptualized the study, and drafted and reviewed the manuscript. BS and XF collected the data. ZZ, JL, and YW analyzed the data. All authors have read and approved the final draft.

## Conflict of Interest

The authors declare that the research was conducted in the absence of any commercial or financial relationships that could be construed as a potential conflict of interest.

## Publisher's Note

All claims expressed in this article are solely those of the authors and do not necessarily represent those of their affiliated organizations, or those of the publisher, the editors and the reviewers. Any product that may be evaluated in this article, or claim that may be made by its manufacturer, is not guaranteed or endorsed by the publisher.
